# (*E*)-5-(3,5-Dimethyl­phen­yl)-*N*-[4-(methyl­sulfan­yl)benzyl­idene]-1,3,4-thia­diazol-2-amine

**DOI:** 10.1107/S1600536810001558

**Published:** 2010-01-20

**Authors:** Jun Hu, Jin-xiu Ji, Ying Zhou, Ji-kui Wang, Yan-hua Xu

**Affiliations:** a Department of Safety Engineering, College of Urban Construction and Safety Engineering, Nanjing University of Technology, Nanjing 210009, People’s Republic of China; b Research & Development Center, Sinochem Jiangsu Corporation, Longpan Road, Nanjing, Nanjing 210002, People’s Republic of China; c Department of Environmental Engineering, College of the Environment, Nanjing University of Technology, Nanjing 210009, People’s Republic of China; d Department of Applied Chemistry, College of Science, Nanjing University of Technology, Nanjing 210009, People’s Republic of China

## Abstract

The title compound, C_18_H_17_N_3_S_2_, was synthesized by the reaction of 5-(3,5-dimethyl­phen­yl)-1,3,4-thia­diazol-2-amine and 4-(methyl­sulfan­yl)benzaldehyde. An intra­molecular C—H⋯S hydrogen bond results in the formation of a planar (r.m.s. deviation = 0.003 Å) five-membered ring. In the crystal structure, inter­molecular C—H⋯N hydrogen bonds link the mol­ecules to form layers parallel to (011).

## Related literature

For the broad spectrum biological activity of 1,3,4-thia­diazole derivatives, see: Nakagawa *et al.* (1996 ▶); Wang *et al.* (1999 ▶).
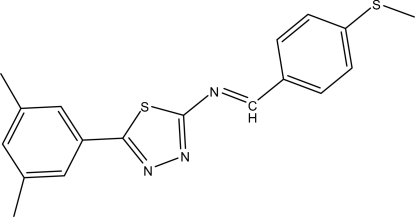


## Experimental

### 

#### Crystal data

C_18_H_17_N_3_S_2_
*M* *_r_* = 339.47
Triclinic, 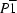

*a* = 8.5640 (17) Å
*b* = 9.3370 (19) Å
*c* = 11.570 (2) Å
α = 90.98 (3)°
β = 110.03 (3)°
γ = 99.66 (3)°
*V* = 854.1 (3) Å^3^
*Z* = 2
Mo *K*α radiation
μ = 0.31 mm^−1^
*T* = 298 K
0.30 × 0.20 × 0.10 mm

#### Data collection

Enraf–Nonius CAD-4 diffractometer
Absorption correction: ψ scan (North *et al.*, 1968 ▶) *T*_min_ = 0.912, *T*_max_ = 0.969
3324 measured reflections
3098 independent reflections
2286 reflections with *I* > 2σ(*I*)
*R*_int_ = 0.031
3 standard reflections every 200 reflections  intensity decay: 1%

#### Refinement

*R*[*F* ^2^ > 2σ(*F* ^2^)] = 0.065
*wR*(*F* ^2^) = 0.208
*S* = 1.00
3098 reflections
209 parameters
H-atom parameters constrained
Δρ_max_ = 0.38 e Å^−3^
Δρ_min_ = −0.46 e Å^−3^

### 

Data collection: *CAD-4 EXPRESS* (Enraf–Nonius, 1994 ▶); cell refinement: *CAD-4 EXPRESS*; data reduction: *XCAD4* (Harms & Wocadlo, 1995 ▶); program(s) used to solve structure: *SHELXS97* (Sheldrick, 2008 ▶); program(s) used to refine structure: *SHELXL97* (Sheldrick, 2008 ▶); molecular graphics: *SHELXTL* (Sheldrick, 2008 ▶); software used to prepare material for publication: *SHELXL97*.

## Supplementary Material

Crystal structure: contains datablocks global, I. DOI: 10.1107/S1600536810001558/bt5165sup1.cif

Structure factors: contains datablocks I. DOI: 10.1107/S1600536810001558/bt5165Isup2.hkl

Additional supplementary materials:  crystallographic information; 3D view; checkCIF report

## Figures and Tables

**Table 1:** Hydrogen-bond geometry (Å, °)

*D*—H⋯*A*	*D*—H	H⋯*A*	*D*⋯*A*	*D*—H⋯*A*
C7—H7*A*⋯N2^i^	0.93	2.58	3.223 (6)	126
C8—H8*A*⋯S2	0.93	2.59	3.041 (5)	110

Symmetry code: (i) 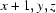.

**Fig. 1.**
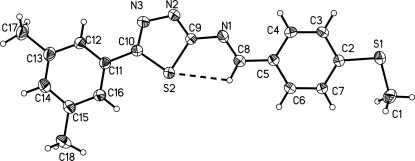
The molecular structure of the title molecule, with the atom-numbering scheme. Displacement ellipsoids are drawn at the 50% probability level. The intramolecular hydrogen bond is shown as a dashed line.

**Fig. 2.**
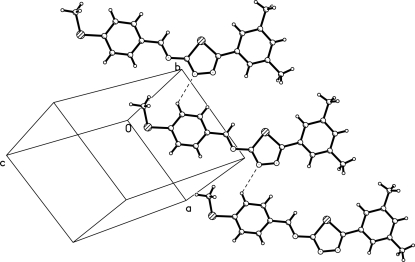
A partial packing diagram of the title compound. Hydrogen bonds are shown as dashed lines.

## supplementary crystallographic information

### Comment

1,3,4-Thiadiazole derivatives represent an interesting class of compounds possessing a broad spectrum biological activities (Nakagawa *et al.*, 1996; Wang *et al.*, 1999). These compounds are known to exhibit diverse biological effects, such as insecticidal, fungicidal activities (Wang *et al.*, 1999).  The molecule (Fig. 1) is almost planar (r.m.s. deviation for all non-H atoms 0.149Å). An intramolecular C—H···N hydrogen bond (Table 1) results in the formation of a planar five-membered ring.   In the crystal structure, intermolecular C—H···N hydrogen bonds (Table 1) link the molecules to form layers parallel to the (0 1 1) plane (Fig. 2).

### Experimental

5-(3,5-dimethylphenyl)-1,3,4-thiadiazol-2-amine(5 mmol) and 4-methylthio benzaldehyde(5 mmol) were added in toluene (50 ml). The water was removed by distillation for 5 h. The reaction mixture was left to cool to room temperature, filtered, and the filter cake was crystallized from acetone to give pure compound (I) (m.p. 408 K).Crystals of (I) suitable for X-ray diffraction were obtained by slow evaporation of an acetone solution.

### Refinement

All H atoms   were placed geometrically at the distances of 0.93–0.97 Å and included in the refinement in riding model approximation with *U*_iso_(H) = 1.2_eq_ of the carrier atom (1.5 for methyl groups).

### Crystal data

**Table:**

C_18_H_17_N_3_S_2_	*Z* = 2
*M**_r_* = 339.47	*F*(000) = 356
Triclinic, *P*1	*D*_x_ = 1.320 Mg m^−^^3^
Hall symbol: -P 1	Melting point: 408 K
*a* = 8.5640 (17) Å	Mo *K*α radiation, λ = 0.71073 Å
*b* = 9.3370 (19) Å	Cell parameters from 25 reflections
*c* = 11.570 (2) Å	θ = 10–13°
α = 90.98 (3)°	µ = 0.31 mm^−^^1^
β = 110.03 (3)°	*T* = 298 K
γ = 99.66 (3)°	Block, colorless
*V* = 854.1 (3) Å^3^	0.30 × 0.20 × 0.10 mm

### Data collection

**Table:**

Enraf–Nonius CAD-4 diffractometer	2286 reflections with *I* > 2σ(*I*)
Radiation source: fine-focus sealed tube	*R*_int_ = 0.031
graphite	θ_max_ = 25.3°, θ_min_ = 1.9°
ω/2θ scans	*h* = 0→10
Absorption correction: ψ scan (North *et al.*, 1968)	*k* = −11→11
*T*_min_ = 0.912, *T*_max_ = 0.969	*l* = −13→13
3324 measured reflections	3 standard reflections every 200 reflections
3098 independent reflections	intensity decay: 1%

### Refinement

**Table:**

Refinement on *F*^2^	Secondary atom site location: difference Fourier map
Least-squares matrix: full	Hydrogen site location: inferred from neighbouring sites
*R*[*F*^2^ > 2σ(*F*^2^)] = 0.065	H-atom parameters constrained
*wR*(*F*^2^) = 0.208	*w* = 1/[σ^2^(*F*_o_^2^) + (0.1*P*)^2^ + 1.550*P*] where *P* = (*F*_o_^2^ + 2*F*_c_^2^)/3
*S* = 1.00	(Δ/σ)_max_ < 0.001
3098 reflections	Δρ_max_ = 0.38 e Å^−^^3^
209 parameters	Δρ_min_ = −0.46 e Å^−^^3^
0 restraints	Extinction correction: *SHELXL97* (Sheldrick, 2008), Fc^*^=kFc[1+0.001xFc^2^λ^3^/sin(2θ)]^-1/4^
Primary atom site location: structure-invariant direct methods	Extinction coefficient: 0.017 (4)

### Special details

**Table:**

Geometry. All e.s.d.'s (except the e.s.d. in the dihedral angle between two l.s. planes) are estimated using the full covariance matrix. The cell e.s.d.'s are taken into account individually in the estimation of e.s.d.'s in distances, angles and torsion angles; correlations between e.s.d.'s in cell parameters are only used when they are defined by crystal symmetry. An approximate (isotropic) treatment of cell e.s.d.'s is used for estimating e.s.d.'s involving l.s. planes.
Refinement. Refinement of *F*^2^ against ALL reflections. The weighted *R*-factor *wR* and goodness of fit *S* are based on *F*^2^, conventional *R*-factors *R* are based on *F*, with *F* set to zero for negative *F*^2^. The threshold expression of *F*^2^ > σ(*F*^2^) is used only for calculating *R*-factors(gt) *etc*. and is not relevant to the choice of reflections for refinement. *R*-factors based on *F*^2^ are statistically about twice as large as those based on *F*, and *R*- factors based on ALL data will be even larger.

### Fractional atomic coordinates and isotropic or equivalent isotropic displacement parameters (Å^2^)

**Table:**

	*x*	*y*	*z*	*U*_iso_*/*U*_eq_	
S1	0.62029 (16)	1.18993 (14)	−0.40284 (12)	0.0614 (4)	
S2	0.10286 (13)	0.70508 (13)	0.07048 (10)	0.0522 (4)	
N1	0.0950 (5)	0.8908 (4)	−0.1158 (3)	0.0502 (9)	
N2	−0.1485 (5)	0.8040 (5)	−0.0728 (4)	0.0692 (12)	
N3	−0.2085 (5)	0.7183 (5)	0.0023 (4)	0.0685 (12)	
C1	0.7907 (6)	1.0978 (6)	−0.3883 (5)	0.0723 (15)	
H1B	0.8553	1.1424	−0.4359	0.108*	
H1C	0.7470	0.9974	−0.4181	0.108*	
H1D	0.8620	1.1039	−0.3031	0.108*	
C2	0.5192 (5)	1.0955 (4)	−0.3116 (4)	0.0445 (9)	
C3	0.3640 (6)	1.1315 (5)	−0.3168 (4)	0.0533 (11)	
H3B	0.3206	1.2023	−0.3674	0.064*	
C4	0.2770 (5)	1.0636 (5)	−0.2484 (4)	0.0524 (11)	
H4A	0.1754	1.0897	−0.2519	0.063*	
C5	0.3370 (5)	0.9560 (4)	−0.1734 (4)	0.0442 (9)	
C6	0.4919 (5)	0.9225 (5)	−0.1672 (4)	0.0487 (10)	
H6A	0.5357	0.8527	−0.1155	0.058*	
C7	0.5814 (5)	0.9899 (4)	−0.2356 (4)	0.0473 (10)	
H7A	0.6838	0.9646	−0.2309	0.057*	
C8	0.2444 (5)	0.8767 (5)	−0.1040 (4)	0.0480 (10)	
H8A	0.2960	0.8130	−0.0488	0.058*	
C9	0.0130 (5)	0.8098 (4)	−0.0484 (4)	0.0447 (9)	
C10	−0.0942 (5)	0.6600 (4)	0.0806 (4)	0.0453 (10)	
C11	−0.1302 (5)	0.5649 (4)	0.1713 (4)	0.0443 (9)	
C12	−0.2857 (5)	0.5491 (5)	0.1857 (4)	0.0491 (10)	
H12A	−0.3676	0.5975	0.1361	0.059*	
C13	−0.3218 (6)	0.4629 (5)	0.2724 (4)	0.0543 (11)	
C14	−0.2009 (6)	0.3892 (5)	0.3429 (4)	0.0580 (12)	
H14A	−0.2254	0.3293	0.4003	0.070*	
C15	−0.0418 (6)	0.4016 (5)	0.3309 (4)	0.0521 (11)	
C16	−0.0071 (5)	0.4910 (5)	0.2446 (4)	0.0484 (10)	
H16A	0.0979	0.5018	0.2356	0.058*	
C17	−0.4903 (7)	0.4480 (6)	0.2888 (6)	0.0737 (15)	
H17A	−0.4931	0.3842	0.3525	0.111*	
H17B	−0.5785	0.4083	0.2128	0.111*	
H17C	−0.5070	0.5421	0.3116	0.111*	
C18	0.0868 (7)	0.3170 (6)	0.4057 (5)	0.0736 (15)	
H18A	0.1873	0.3396	0.3853	0.110*	
H18B	0.0416	0.2146	0.3877	0.110*	
H18C	0.1137	0.3426	0.4919	0.110*	

### Atomic displacement parameters (Å^2^)

**Table:**

	*U*^11^	*U*^22^	*U*^33^	*U*^12^	*U*^13^	*U*^23^
S1	0.0633 (8)	0.0675 (8)	0.0680 (8)	0.0195 (6)	0.0371 (6)	0.0291 (6)
S2	0.0423 (6)	0.0626 (7)	0.0570 (7)	0.0146 (5)	0.0209 (5)	0.0229 (5)
N1	0.059 (2)	0.049 (2)	0.052 (2)	0.0151 (17)	0.0275 (17)	0.0160 (16)
N2	0.056 (2)	0.087 (3)	0.079 (3)	0.031 (2)	0.032 (2)	0.048 (2)
N3	0.050 (2)	0.088 (3)	0.080 (3)	0.030 (2)	0.029 (2)	0.045 (2)
C1	0.064 (3)	0.099 (4)	0.074 (3)	0.026 (3)	0.043 (3)	0.021 (3)
C2	0.044 (2)	0.044 (2)	0.049 (2)	0.0095 (17)	0.0200 (18)	0.0111 (18)
C3	0.056 (3)	0.056 (3)	0.059 (3)	0.026 (2)	0.026 (2)	0.028 (2)
C4	0.045 (2)	0.053 (3)	0.067 (3)	0.020 (2)	0.024 (2)	0.017 (2)
C5	0.043 (2)	0.042 (2)	0.051 (2)	0.0098 (17)	0.0194 (18)	0.0093 (18)
C6	0.049 (2)	0.047 (2)	0.054 (2)	0.0168 (19)	0.0191 (19)	0.0185 (19)
C7	0.041 (2)	0.048 (2)	0.059 (3)	0.0137 (18)	0.0221 (19)	0.017 (2)
C8	0.049 (2)	0.050 (2)	0.048 (2)	0.0151 (19)	0.0191 (19)	0.0134 (19)
C9	0.040 (2)	0.047 (2)	0.052 (2)	0.0128 (17)	0.0206 (18)	0.0145 (19)
C10	0.047 (2)	0.045 (2)	0.049 (2)	0.0142 (18)	0.0204 (19)	0.0084 (18)
C11	0.045 (2)	0.041 (2)	0.051 (2)	0.0073 (17)	0.0222 (19)	0.0070 (18)
C12	0.046 (2)	0.047 (2)	0.061 (3)	0.0122 (18)	0.025 (2)	0.012 (2)
C13	0.055 (3)	0.049 (2)	0.066 (3)	0.006 (2)	0.032 (2)	0.007 (2)
C14	0.066 (3)	0.051 (3)	0.062 (3)	0.010 (2)	0.029 (2)	0.018 (2)
C15	0.056 (3)	0.049 (2)	0.052 (2)	0.010 (2)	0.020 (2)	0.012 (2)
C16	0.044 (2)	0.049 (2)	0.054 (2)	0.0114 (18)	0.0170 (19)	0.0092 (19)
C17	0.070 (3)	0.073 (3)	0.096 (4)	0.009 (3)	0.053 (3)	0.015 (3)
C18	0.084 (4)	0.075 (3)	0.067 (3)	0.033 (3)	0.023 (3)	0.031 (3)

### Geometric parameters (Å, °)

**Table:**

S1—C2	1.745 (4)	C6—H6A	0.9300
S1—C1	1.777 (5)	C7—H7A	0.9300
S2—C10	1.714 (4)	C8—H8A	0.9300
S2—C9	1.736 (4)	C10—C11	1.465 (6)
N1—C8	1.269 (5)	C11—C12	1.382 (6)
N1—C9	1.376 (5)	C11—C16	1.399 (6)
N2—C9	1.305 (5)	C12—C13	1.380 (6)
N2—N3	1.360 (5)	C12—H12A	0.9300
N3—C10	1.290 (5)	C13—C14	1.375 (6)
C1—H1B	0.9600	C13—C17	1.503 (6)
C1—H1C	0.9600	C14—C15	1.403 (6)
C1—H1D	0.9600	C14—H14A	0.9300
C2—C7	1.384 (6)	C15—C16	1.389 (6)
C2—C3	1.407 (6)	C15—C18	1.500 (6)
C3—C4	1.358 (6)	C16—H16A	0.9300
C3—H3B	0.9300	C17—H17A	0.9600
C4—C5	1.387 (6)	C17—H17B	0.9600
C4—H4A	0.9300	C17—H17C	0.9600
C5—C6	1.392 (5)	C18—H18A	0.9600
C5—C8	1.442 (5)	C18—H18B	0.9600
C6—C7	1.371 (6)	C18—H18C	0.9600
			
C2—S1—C1	103.0 (2)	N1—C9—S2	126.2 (3)
C10—S2—C9	86.66 (19)	N3—C10—C11	122.6 (4)
C8—N1—C9	119.2 (4)	N3—C10—S2	114.3 (3)
C9—N2—N3	112.4 (4)	C11—C10—S2	123.1 (3)
C10—N3—N2	113.2 (4)	C12—C11—C16	119.6 (4)
S1—C1—H1B	109.5	C12—C11—C10	120.0 (4)
S1—C1—H1C	109.5	C16—C11—C10	120.3 (4)
H1B—C1—H1C	109.5	C13—C12—C11	121.3 (4)
S1—C1—H1D	109.5	C13—C12—H12A	119.4
H1B—C1—H1D	109.5	C11—C12—H12A	119.4
H1C—C1—H1D	109.5	C14—C13—C12	118.7 (4)
C7—C2—C3	118.8 (4)	C14—C13—C17	120.4 (4)
C7—C2—S1	124.6 (3)	C12—C13—C17	120.9 (4)
C3—C2—S1	116.6 (3)	C13—C14—C15	121.9 (4)
C4—C3—C2	120.5 (4)	C13—C14—H14A	119.1
C4—C3—H3B	119.8	C15—C14—H14A	119.1
C2—C3—H3B	119.8	C16—C15—C14	118.5 (4)
C3—C4—C5	121.1 (4)	C16—C15—C18	120.2 (4)
C3—C4—H4A	119.4	C14—C15—C18	121.3 (4)
C5—C4—H4A	119.4	C15—C16—C11	120.0 (4)
C4—C5—C6	118.1 (4)	C15—C16—H16A	120.0
C4—C5—C8	122.7 (4)	C11—C16—H16A	120.0
C6—C5—C8	119.2 (4)	C13—C17—H17A	109.5
C7—C6—C5	121.5 (4)	C13—C17—H17B	109.5
C7—C6—H6A	119.2	H17A—C17—H17B	109.5
C5—C6—H6A	119.2	C13—C17—H17C	109.5
C6—C7—C2	120.0 (4)	H17A—C17—H17C	109.5
C6—C7—H7A	120.0	H17B—C17—H17C	109.5
C2—C7—H7A	120.0	C15—C18—H18A	109.5
N1—C8—C5	122.3 (4)	C15—C18—H18B	109.5
N1—C8—H8A	118.9	H18A—C18—H18B	109.5
C5—C8—H8A	118.9	C15—C18—H18C	109.5
N2—C9—N1	120.4 (4)	H18A—C18—H18C	109.5
N2—C9—S2	113.3 (3)	H18B—C18—H18C	109.5
			
C9—N2—N3—C10	−0.7 (7)	C10—S2—C9—N1	−179.3 (4)
C1—S1—C2—C7	8.1 (5)	N2—N3—C10—C11	179.3 (4)
C1—S1—C2—C3	−172.1 (4)	N2—N3—C10—S2	0.1 (6)
C7—C2—C3—C4	0.0 (7)	C9—S2—C10—N3	0.3 (4)
S1—C2—C3—C4	−179.8 (4)	C9—S2—C10—C11	−178.9 (4)
C2—C3—C4—C5	−1.0 (7)	N3—C10—C11—C12	−8.1 (7)
C3—C4—C5—C6	1.9 (7)	S2—C10—C11—C12	171.1 (3)
C3—C4—C5—C8	−177.0 (4)	N3—C10—C11—C16	172.7 (4)
C4—C5—C6—C7	−1.9 (7)	S2—C10—C11—C16	−8.2 (6)
C8—C5—C6—C7	177.0 (4)	C16—C11—C12—C13	0.8 (7)
C5—C6—C7—C2	1.0 (7)	C10—C11—C12—C13	−178.5 (4)
C3—C2—C7—C6	0.0 (7)	C11—C12—C13—C14	−1.6 (7)
S1—C2—C7—C6	179.8 (3)	C11—C12—C13—C17	179.1 (4)
C9—N1—C8—C5	178.8 (4)	C12—C13—C14—C15	1.3 (7)
C4—C5—C8—N1	6.6 (7)	C17—C13—C14—C15	−179.4 (4)
C6—C5—C8—N1	−172.3 (4)	C13—C14—C15—C16	−0.2 (7)
N3—N2—C9—N1	179.6 (4)	C13—C14—C15—C18	−178.1 (5)
N3—N2—C9—S2	0.9 (6)	C14—C15—C16—C11	−0.7 (7)
C8—N1—C9—N2	−168.9 (4)	C18—C15—C16—C11	177.2 (4)
C8—N1—C9—S2	9.7 (6)	C12—C11—C16—C15	0.4 (6)
C10—S2—C9—N2	−0.7 (4)	C10—C11—C16—C15	179.7 (4)

### Hydrogen-bond geometry (Å, °)

**Table:**

*D*—H···*A*	*D*—H	H···*A*	*D*···*A*	*D*—H···*A*
C7—H7A···N2^i^	0.93	2.58	3.223 (6)	126
C8—H8A···S2	0.93	2.59	3.041 (5)	110

Symmetry codes: (i) *x*+1, *y*, *z*.
